# Supplementary material for: Implementation fidelity of tuberculosis screening for Diabetes mellitus patients among healthcare providers offering diabetes services in Ubungo District, Dar es Salaam, Tanzania
Source: PLOS Glob Public Health. 2026 May 12;6(5):e0005768. doi: 10.1371/journal.pgph.0005768 (PMC13166936; doi:10.1371/journal.pgph.0005768)
Supplement: S1 Text — This document contains the full structured questionnaire used to assess provider-level implementation fidelity, based on content and frequency details. The questions are grouped into three domains: screening for TB signs and symptoms, patient education and counseling, and referrals, in accordance with the National TB and Leprosy Program guidelines. (DOCX) [file pgph.0005768.s002.docx]

**Appendix 1: Questionnaire**

Questionnaire on provider-level implementation fidelity of Tuberculosis screening for patients with Diabetes mellitus at public health facilities in Ubungo district, Dar es Salaam region. (English version)

For official use only

ID…………………

Date of filling the questionnaire……………

The questionnaire is divided into three sections: A, B, and C.

Instructions:

Part A consists of social demographic information of healthcare providers, which one is required to complete by selecting a corresponding letter by putting a circle (○) in the right-hand side that has the response category.

2. Part B consists of the healthcare provider-level implementation fidelity of Tuberculosis screening among diabetes patients at public health facilities in Ubungo district, Dar-es-salaam region, based on the details of content and frequency questions, which one is required to complete by putting a circle on a corresponding letter (○)

3. Part C consists of factors affecting provider-level implementation fidelity of Tuberculosis screening among DM patients at public health facilities in the Dar-es-salaam region, and one will be required to complete it by putting a circle (○) on a corresponding letter.

Section A: Social Demographic information of healthcare providers:

| S/N | VARIABLE | RESPONSE |
| --- | --- | --- |
| 1. | Age | A.18-34 |
| 2. | Gender | B.35-60 |
| 3. | Level of education | A. Degree and above  B. Diploma and below |
| 4. | Level of facility | A. Hospital  B.Health center  C.Dispensary |
| 5. | Years working in a facility | A. 1 to 5 years  B.Above 5 years |

**Questions about the provider-level implementation fidelity of TB screening among DM patients, based on the content details.**

**Content questions:**

| S/N | QUESTION | RESPONSE |
| --- | --- | --- |
| **signs& symptoms questions** | | |
| 1. | Do you ask the DM patients about a cough for two weeks or more during consultation? | A. Yes  B. No |
| 2. | Do you ask the DM patients if they have excessive night sweats during consultation? | A. Yes  B. No |
| 3. | Do you ask the DM patients if they have had a fever during consultation? | A. Yes  B. No |
| 4. | Do you ask the DM patients if they have chest pain and cough up blood during consultation? | A. Yes  B. No |
| 5. | Do you ask for noticeable weight loss or 3 kg weight loss in a month of DM patients during consultation? | A. Yes  B. No |
| **Education& counselling questions** | | |
| 6 | Do you provide tuberculosis education to patients with diabetes? | A. Yes  B. No |
| 7 | Do you provide counseling on tuberculosis screening for patients with diabetes? | A. Yes  B. No |
|  | **Referral question** | |
| 8 | Do you refer diabetic patients who show positive signs of tuberculosis infection to a TB clinic for a sputum sample  collection for diagnosis and treatment? | A. Yes  B. No |

**Questions on the provider-level implementation fidelity of TB screening among DM patients based on the details of frequency.**

**Frequency questions:**

| S/N | QUESTION | RESPONSE |
| --- | --- | --- |
| **Signs symptoms questions** | | |
| 9. | How frequently do you ask DM patients about cough for two weeks or more during consultation? | A. Never  B. Rarely  C.For all new clients  D. For Most clients  E. All the time for all clients |
| 10. | How frequently do you ask DM patients about excessive night sweats of any duration during consultation? | A. Never  B. Rarely  C.For all new clients  D. For Most clients  E. All the time for all clients |
| 11. | How frequently do you ask DM patients if they had a fever during consultation? | A. Never  B. Rarely  C.For all new clients  D. For Most clients  E. All the time for all clients |
| 12. | How frequently do you ask DM patients about chest pain and coughing up blood during consultation? | A. Never  B. Rarely  C.For all new clients  D. For Most clients  E. All the time for all clients |
| 13. | How frequently do you ask/check for noticeable weight loss or a 3 kg weight loss in a month of DM patients during consultation? | A. Never  B. Rarely  C.For all new clients  D. For Most clients  E. All the time for all clients |
|  | **Education &counselling question** | |
| 14. | How frequently do you provide TB education for patients with DM? | A. Never  B. Rarely  C.For all new clients  D. For Most clients  E. All the time for all clients |
| 15. | How frequently do you provide counselling about TB screening for patients with DM? | A. Never  B. Rarely  C.For all new clients  D. For Most clients  E. All the time for all clients |
| **Referral question** | | |
| 16. | How frequently do you refer diabetic patients who show positive signs of tuberculosis infection to a TB clinic for  diagnosis and treatment? | A. Never  B. Rarely  C.For all new clients  D. For Most clients  E. All the time for all clients |

**Factors affecting provider-level implementation fidelity of TB screening among DM patients at public health facilities offering DM services**.

System-related factors: We have provided several statements and questions below. For either statement or question, please indicate by putting a circle (○) among the given answers in the category section. There is no right or wrong answer; please indicate how you feel.

| **Teamwork** | | |
| --- | --- | --- |
| 17. | Do you agree that having good teamwork, which includes good communication and networking, both formal and non-formal, among staff, influences the adherence towards screening of TB for DM patients as per the National TB guideline? | A. Strongly disagree  B. Disagree  C.Neutral  D.Agree  E. Strongly agree |
| **Training** | | |
| 18. | Do you agree that having training on TB screening for DM patients in your facility or outside your facility will influence its adherence as per what is recommended in the National TB guideline? | A. Strongly disagree  B. Disagree  C. Neutral  D.Agree  E. Strongly agree |
| **Staff allocation** | | |
| 19. | Do you agree that having adequate staff in your facility to perform TB screening for DM patients influences adherence towards National TB guideline, which recommends it? | A. Strongly disagree  B. Disagree  C. Neutral  D.Agree  E. Strongly agree |
| **Records and Documentations** | | |
| 20. | Do you agree that keeping records and documents in your facility for DM patients screened for TB influences adherence to the National TB guideline that recommends it? | A. Strongly disagree  B. Disagree  C. Neutral  D.Agree  E. Strongly agree |

Providers -related factors

We have provided several questions below. For each question, please respond by putting a circle (○) among the given answers in the category section. There is no right or wrong answer; please indicate how you feel.

| **Familiarity with the National TB guideline** | | |
| --- | --- | --- |
| 21 | How familiar are you with the screening of TB for DM patients as per the National TB guideline contents? | A. Very poor  B. Poor  C.Fair  D.Good  E. Very good |
| **Self-efficacy** | | |
| 22 | How do you grade yourself on Self-efficacy as a health provider with enough capabilities to execute screening of TB for DM patients, as per the National TB guideline? | A. Very poor  B. Poor  C.Fair  D.Good  E. Very good |

Guideline-related factors

Concerning the TB screening among DM patients as per the National TB guideline, for each question, please respond by putting a circle (○) among the given answers in the category section. There is no right or wrong answer; please indicate how you feel.

| **Nature and source of the National TB guideline** | | |
| --- | --- | --- |
| 23 | Do you agree that knowing the nature and source of TB screening for DM patients, which comes from the National TB guideline, influences its adherence? | A. Strongly disagree  B. Disagree  C. Neutral  D.Agree  E. Strongly agree |
|  |  |  |
|  | **Relative advantage** | |
| 24 | Do you agree that the adherence to TB screening for DM patients as per the National TB guideline has a relative advantage compared to the use of other tools? | A. Strongly disagree  B. Disagree  C. Neutral  D.Agree  E. Strongly agree |
| **Design quality and package** | | |
| 25 | Do you agree that the directives are designed, bundled, presented, and assembled on TB screening for DM patients from the National TB guideline, which influences their adherence? | A. Strongly disagree  B. Disagree  C. Neutral  D.Agree  E. Strongly agree |
