# Supplementary material for: Implementation fidelity of tuberculosis screening for Diabetes mellitus patients among healthcare providers offering diabetes services in Ubungo District, Dar es Salaam, Tanzania
Source: PLOS Glob Public Health. 2026 May 12;6(5):e0005768. doi: 10.1371/journal.pgph.0005768 (PMC13166936; doi:10.1371/journal.pgph.0005768)
Supplement: Attachment — Submitted filename: Plos Global Public Health Reviewers response matrix.docx [file pgph.0005768.s004.docx]

**RESPONSE MATRIX TO REVIEWERS COMMENTS**

**Title:** Implementation Fidelity of Tuberculosis Screening for Diabetes Mellitus Patients among healthcare providers offering Diabetes services in Ubungo District, Dar es Salaam, Tanzania.

**Manuscript ID:** PGPH-D-25-03950

**Authors:** Edwin Christian Chavala.

Felistar Mwakasungura

Linda Simon Paulo

Tumaini Nyamhanga

| **Section** | **Journal requirements** | **Author responses** | **Page Lines. where changes have been made** |
| --- | --- | --- | --- |
| **Ethics Statement** | Please ensure that your Ethics Statement is available in its entirety at the beginning of your Methods section, under a subheading 'Ethics Statement'. | I have added a full Ethics Statement subheading at the start of the Methods section, detailing institutional review board approval, informed consent procedures, and compliance with ethical guidelines. | Page 6, Lines 90-95 |
| **Title** | Please ensure that the Title in your manuscript file and the Title provided in your online submission form are the same. | The title in the manuscript file matches the submission form exactly | Page 1, Lines 1-3 |
| **Supporting Information** | We have noticed that you have uploaded Supporting Information files, but you have not included a list of legends. Please add a full list of legends for your Supporting Information files after the references list. | I have added a complete list of legends for all Supporting Information files (S1 Fig: Provider-level implementation fidelity of TB screening among DM patients by facility level; S1 Text: Questionnaire on provider-level implementation fidelity based on the details of content and frequency. | Page 36, Lines 584-594 |
| **Data Availability** | For studies involving third-party data, we encourage authors to share any data specific to the analyses that they can legally distribute. PLOS recognizes, however, that authors may be using third-party data that they do not have the right to share. When third-party data cannot be publicly shared, authors must provide all information necessary for interested researchers to apply to gain access to the data. (https://journals.plos.org/plosone/s/data-availability#loc-acceptable-data-access-restrictions) | The Data Availability Statement has been revised in the PLOS system to clarify that de-identified study data are available upon request with MUHAS IRB approval, and that aggregate DHIS2 data are restricted by government policy, with guidance provided for access applications. |  |
|  | **Comments from the Reviewers** |  |  |
| **Overall** | **Reviewer 1:** Discussion could be strengthened by drawing clearer implications for policy and scalability, and by explaining how lessons from high-fidelity settings can be adapted to low-fidelity settings. Adding explanatory footnotes to some of the tables and ensuring that figures and tables, supporting materials are properly referenced in the text | The Discussion section has been restructured and expanded with new paragraphs that clearly outline policy implications and scalability  Explanatory footnotes have been added to all relevant tables (e.g., Table 7&8 now include a footnote defining (aPR) as “adjusted prevalence ratio” and (CI) as confidence intervals. All clarifying figures, tables, and supporting materials are now explicitly referenced in the text where they are discussed (e.g., S1 Fig for provider-level IF and S1 Text. Questionnaire on provider-level IF of TB screening described in the Results and Discussion sections). | Page 26, Lines 374-420 |
|  | **Reviewer 2:**This manuscript addresses an important and understudied implementation science topic: implementation fidelity of tuberculosis (TB) screening among diabetes mellitus (DM) patients in routine care settings in Tanzania. The topic is relevant to TB–DM collaborative activities and aligns well with global priorities. However, several substantive issues need to be addressed before the findings can be interpreted with confidence. |  |  |
| **Sampling strategy** | There is a lack of clarity and internal consistency between the sampling strategy described in the Methods and the way provider numbers are reported in the Results. The authors should clearly report the number of participants recruited per facility, ideally in a supplementary table, and explain how the stated sampling strategy was operationalised. | I determined the sample size using Cochran's formula for infinite populations, then applied the finite population correction since the total number of healthcare providers offering diabetes mellitus (DM) services across the 20 public health facilities in Ubungo District was known (N = 168). The final sample size was calculated as 94 providers. Proportionate stratified sampling was then used to allocate the required number of participants to each facility level (hospitals, health centers, and dispensaries) based on the proportion of providers in each stratum. The number of providers sampled per facility type is shown in Table 2.  . | Page 8, Lines150-166 |
| **Statistical reporting** | The analytical approach (modified Poisson regression) is appropriate for the outcome, but there appear to be reporting errors (e.g., confidence intervals in Tables 7 and 8, where the bounds appear inconsistent). These should be carefully checked. In addition, typographical errors (e.g., “modified poison regression”) should be corrected. | I have corrected all confidence intervals in Tables 7 and 8. Typographical errors (e.g., "poison" to "Poisson") have been fixed throughout. | Page22&24, |
| **Outcome definition and interpretation** | The primary outcome is provider-level implementation fidelity, measured through self-reported adherence to TB screening guideline components. However, the Results and Discussion repeatedly imply patient-level screening coverage (e.g., statements suggesting that a certain proportion of DM patients were screened for TB). No patient-level numerator or denominator is presented, and the Methods do not describe record review or observation. The authors should consistently frame the outcome as provider-level fidelity, revise language that implies patient screening coverage, and explicitly acknowledge the absence of patient-level screening data as a limitation if such data were not collected. | I have revised all language to frame the outcome strictly as provider-level implementation fidelity  Added to the limitation section on the absence of patient-level data (no observation). | Page 16-26 |
| **Results(Tables)** | Adding explanatory footnotes to some of the tables and ensuring that figures and tables, supporting materials are properly referenced in the text. | As noted in response to Reviewer 1 have added footnotes (e.g., defining abbreviations like aPR, CI, and Ref to all tables and ensuring in-text references (e.g., S1 Fig for fidelity by facility level)) and | Page 22&24-for tables.  Page 20-for S1figure |
| **Discussion focus** | The Discussion begins by restating the study’s aim and strengths rather than clearly summarising the key findings. Several paragraphs repeat results or focus heavily on comparisons with other studies, with limited interpretation of what the findings mean for the Ubungo or Tanzanian primary care context. The Discussion would be strengthened by focusing on (i) the most poorly implemented screening components, (ii) why dispensaries showed lower fidelity, and (iii) the implications for TB–DM integration, supervision, and training in similar settings. | I have restructured the discussion to start with a summary of key findings. I also reduced repetition of putting much focus on comparisons and instead added focus on poor components, such as education/counseling, and dispensaries' low fidelity, and what the findings imply. | Page 26 |
| **Unsupported causal explanations** | The Discussion attributes low implementation fidelity (17%) to factors such as lack of integrated TB–DM training and provider role allocation, yet these explanations are not adequately supported by the study data. Training does not appear to remain significant in adjusted analyses, and several explanatory statements are not referenced. In addition, the Discussion suggests that degree-holding providers may focus on administrative duties, while the Methods state that staff in administrative roles were excluded from the study. These contradictions should be resolved, and causal language should be softened or removed where not directly supported by evidence. | I have removed direct attribution of low fidelity (17%) to unsupported factors like lack of integrated TB-DM training and explicitly noted training's non-significance in adjusted analyses.  Added references to all explanatory statements (e.g., gaps in education/counseling.  Completely removed any suggestion that degree-holding providers focus on administrative duties, resolving the contradiction with the Methods  Replaced strong causal language throughout with softened phrases and removed it in areas not supported with evidence.  . | Page 26 |
| **Limitations section** | The limitations are acknowledged; however, they could be more clearly framed from an implementation science perspective, including reliance on self-reported practices, absence of observational or record-based verification, and the cross-sectional design limiting causal inference. | I have expanded the limitations section with implementation science framing (e.g., self-report bias, lack of observation/records, cross-sectional studies' limitations on causality). | Page 28, Lines 422-429 |
