# Supplementary material for: Implementation fidelity of tuberculosis screening for Diabetes mellitus patients among healthcare providers offering diabetes services in Ubungo District, Dar es Salaam, Tanzania
Source: PLOS Glob Public Health. 2026 May 12;6(5):e0005768. doi: 10.1371/journal.pgph.0005768 (PMC13166936; doi:10.1371/journal.pgph.0005768)
Supplement: Attachment — Submitted filename: PLOS GLOBAL PUBLIC HEALTH JOURNAL REVIEWERS RESPONSE MATRIX.docx [file pgph.0005768.s005.docx]

**RESPONSE MATRIX TO REVIEWERS COMMENTS**

**Title:** Implementation Fidelity of Tuberculosis Screening for Diabetes Mellitus Patients among healthcare providers offering Diabetes services in Ubungo District, Dar es Salaam, Tanzania.

**Manuscript ID:** PGPH-D-25-03950

**Authors:** Edwin Christian Chavala.

Felistar Mwakasungura

Linda Simon Paulo

Tumaini Nyamhanga

| **Section** | **Journal requirements** | **Author responses** | **Page Lines. where changes have been made** |
| --- | --- | --- | --- |
| **Data Availability** | Concerns about whether the data underlying the findings are fully available. | I confirm that all underlying data are publicly available via Figshare (DOI provided in the manuscript). The dataset includes all variables used in the analysis.  I have clarified the Data Availability Statement to explicitly indicate that the dataset contains the full provider-level data used to generate the reported findings. | Page 30, Lines 465-467 |
| **Comments from the Reviewers** | | | |
|  |  |  |  |
| **Overall** | **Reviewer 2.** |  |  |
| **Sampling strategy** | Clarify whether clustering by facility was considered analytically or acknowledge this as a limitation. | I have now explicitly acknowledged this as a limitation in the revised manuscript.  . | Page 29, Lines 445- 447 |
| **Statistical reporting** | In Table 8, “Design quality” is reported with an adjusted PR of 1.43 and a 95% CI of 0.67–1.37, which is not possible because the point estimate lies outside the confidence interval | Design quality in regression tables (Table 8) has been thoroughly rechecked and corrected. | Page 26 |
| **Outcome definition and interpretation** | The manuscript should still be checked carefully to ensure that no wording implies patient-level screening coverage, because the study was based on provider self-report rather than patient-level numerator/denominator data, record review, or observation | I have carefully reviewed the manuscript and revised wording throughout to consistently reflect provider-level implementation fidelity, avoiding any implication of patient-level screening coverage. | Page 17, Lines 292- 294  Page 21, Lines 314- 316 |
| **Discussion&Conclussion** | The recommendations go beyond what was directly demonstrated in the adjusted analyses. These points may be reasonable implementation implications, but they should be framed more cautiously. | I agree and have revised and moderated the language in the Discussion and Conclusion sections, which have now been rephrased as cautious suggestions to remain aligned with the data, with potential further research implications added. | Page 29, Lines 435- 458 |
| **Limitations section** | The limitations section cites reference 41 to support the lack of observational or record-based verification, yet reference 41 is a paper on the International Court of Justice and is clearly unrelated to implementation fidelity or health research methods. These issues raise concern that the reference list has not yet been fully checked for citation-to-claim matching, completeness, and use of authoritative sources. | Reference 41 has been replaced with an appropriate methodological reference on the limitations of self-reported data and the value of direct observation in implementation research. | Page 29, Lines 444-445 |
| **Referrence** | The manuscript cites a Google search URL as reference 2 for the WHO global TB burden statement, which is not an acceptable bibliographic source for such a key claim | I have also performed a full audit of the reference list for accuracy, relevance, and proper formatting. Reference 2 now aligns with the claims it supports, and we removed or replaced any unreliable sources. | Page 31, Lines 477-480 |
